# Agenesis of Multiple Primary Teeth and

**DOI:** 10.5005/jp-journals-10005-1188

**Published:** 2013-04-26

**Authors:** SVSG Nirmala, Niraj Gokhale, N Sivakumar, Md Akhil Quadar

**Affiliations:** Professor, Department of Pedodontics and Preventive Dentistry Narayana Dental College, Nellore, Andhra Pradesh-524002, India; Senior Lecturer, Department of Pedodontics and Preventive Dentistry Maratha Mandal Institute of Dental Sciences, Belgaum, Karnataka India; Professor and Head, Professor and Head, Department of Pedodontics and Preventive Dentistry, Narayana Dental College, Nellore, Andhra Pradesh, India; Postgraduate Student, Department of Pedodontics and Preventive Dentistry, Narayana Dental College, Nellore, Andhra Pradesh, India

**Keywords:** Agenesis, Nonsyndromic, Oligodontia, Primary dentition

## Abstract

Agenesis of multiple primary teeth is rare. A 6-year-old girl visited our department, in the absence of any other systemic abnormalities, on clinical and radiographic evaluation, it was revealed that she had only 6 primary teeth in her mouth. Maxillary and mandibular removable partial dentures were fabricated for prosthodontic rehabilitation. At the 6 months follow-up nutrition of the patient as well as self-confident appearance was improved.

**How to cite this article:** Nirmala SVSG, Gokhale N, Sivakumar N, Quadar MA. Agenesis of Multiple Primary Teeth and Its Rehabilitation: A Case Report. Int J Clin Pediatr Dent 2013; 6(1):55-57.

## INTRODUCTION

Agenesis is rare in the primary dentition.^1,2^ Indeed, the reduction in number of teeth is concomitant with reduction in the size of jaw in human evolution and believed to be a continuing evolutionary trend. Stewart defined hypodontia as absence of one or few teeth, oligodontia as agenesis of numerous teeth (more than six) and complete absence of teeth as anodontia.^3^ The prevalence of hypodontia in the primary dentition is 0.1 to 0.9% whereas in permanent dentition 2 to 10%.^4-6^ It usually affects maxillary lateral incisors, mandibular central and lateral incisors^7^ with the common missing teeth in Asian population being the mandibular incisors.^8^ Oligodontia of permanent dentition is common and is considered as variant of normal pattern.^9^It can occur alone or be associated with specific syndromes like ectodermal dysplasia or severe systemic abnormalities.^10^

Grahnen in 1956 conducted a classical study on parents and siblings of 171 hypodontia patients and reported that oligodontia is mainly determined by a dominant autosomal gene pattern with incomplete penetrance of the trait and variable expressivity.^11^ Several factors like trauma, infection of developing tooth bud, radiation overdose, glandular dysfunction, systemic conditions like rickets or syphilis, german measles during pregnancy and severe intrauterine disturbances have been proposed as etiological factors.^10^ This article presents a case of 6-year-old child with 14 missing primary teeth with discussion on the management.

## CASE REPORT

**Fig. 1:**
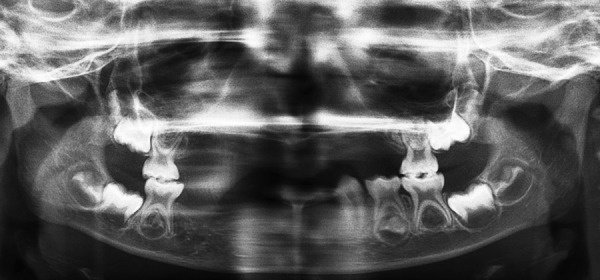
The panoramic radiograph revealed the presence of maxillary and mandibular right and left first permanent molar tooth buds, lower left first and second premolar tooth bud and lower right and left second permanent molar tooth buds. Also it reveals beginning of calcification of lower right second premolar

**Fig. 2:**
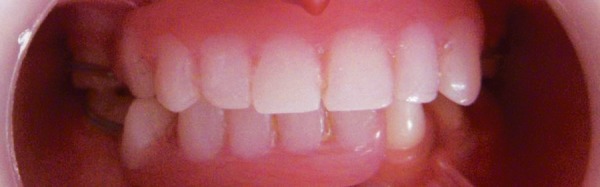
Postoperative intraoral view of the patient with upper and lower removable partial dentures

A 6-year-old asian girl was referred to the Department of Pediatric Dentistry with a chief complaint of several missing teeth. The child was in good health and the health history did not reveal any systemic disease, child was born to non-consanguineous parents. Mother reported uneventful pregnancy and no significant family history. According to the mother no tooth had been lost due to trauma, extraction or exfoliation of teeth for this child. Extraoral examination revealed facial symmetry and no skeletal malocclusion. The child was examined with particular attention to hair, nail, eyes and ears, all of which appeared to be normal. No sweating abnormality was reported by the patient's mother. An intraoral examination revealed the presence of maxillary right and left primary second molars, mandibular right primary second molar, mandibular left primary canine, first and second molars. The teeth were of normal size, shape, and color. The alveolus present was very thin. There were no carious teeth and the patient has good oral hygiene. None of the teeth were mobile. Her oral range of motion was within normal limits. No temporomandibular joint sounds or masticatory muscle pain was noted. Panoramic radiograph revealed congenital absence of 14 primary teeth which included maxillary right and left central incisors, lateral incisors, canines (cuspids), and first primary molars. It also showed absence of right and left lower central incisors, lateral incisors, right canine and right first primary molar and also revealed the presence of all four first permanent molar tooth buds, lower left first and second premolar tooth buds and lower right and left second permanent molar tooth buds. Radiograph showed the beginning of calcification of cusp of lower right second premolar and probably early beginning of calcification of left mandibular second premolar (Fig. 1). The treatment plan included prosthesis in the form of simple acrylic partial dentures and oral health education. Maxillary and mandibular removable partial dentures were inserted with minor occlusal adjustments (Fig. 2). The patient and parent were shown about proper insertion, removal and maintenance of the prosthesis and instructions were given on adequate oral hygiene. Regular recalls were scheduled for 3 months to make necessary adjustments and to monitor the patient's compliance and oral hygiene.

## DISCUSSION

This case is very interesting for several reasons: It reports maximum number of missing primary teeth (n = 14), missing primary first molars, missing permanent tooth buds and absence of any clinical features of the syndromes associated with oligodontia of primary dentition. Ringqvist and Thilander have reported a study with 17 missing teeth in 5,513 children. But the age of the child and type of dentition is not clear from the data given.^12^ Daugaard-Jenen et al have reported one case with 14 missing teeth in their study of 193 cases, but there is no mention as to whether this particular case is of a syndrome associated or not.^13^ Shashikiran et al reported a case of idiopathic oligodontia with nine missing teeth.^14^ Karthik Venkataraghavan et al reported a case of 18 missing teeth, with a history of consanguineous marriage, which could be one of the reasons for the condition as quoted by the authors.^15^ Shilpa et al reported a case of idiopathic oligodontia with 14 missing teeth.^16^ Grahen and Graneth reported that oligodontia in primary dentition always showed aplasia of succedaneous teeth.^5^ The present case also showed the same where maxillary and mandibular anterior succedaneous tooth buds were absent. They may be congenitally missing or may appear later in life. Stones reported a case of absence of maxillary first molar and seven other teeth.^17^ In this case, agenesis was observed of all the first primary molars except mandibular left first primary molar which is very rare. However in Muller's^18^ study of 13,459 children and Haavikko's^19^ of 1,041 children, no cases of absent maxillary first molars were found. In the presented case, three primary first molars were absent which has not been reported earlier. The radiographic evidence did not show any embedded primary molar tooth buds. By the age of 6 years, mandibular central, lateral incisors, canines, first permanent molars, premolars, and second molar tooth buds must be present radiographically.^20^ In the present case all first permanent molar tooth buds, left lower first premolar and lower permanent second molar tooth buds were seen. In this case there was an absence of maxillary right and left primary canines and right mandibular primary canine, but left lower primary canine is present and corresponding succedaneous tooth bud was absent. It could be formed later or it can be congenitally missing and might be a coincidental finding.

There was absence of clinical features of syndromes known to be associated with oligodontia, as the patient did not show any features of syndromes like ectoderemal dysplasia, Reiger's syndrome, Down's syndrome, Lacrimo-auriculo-dento digital syndrome and Marshall's syndrome.^21^ Hence, this is a case of nonsyndromic oligodontia with maximum missing teeth in primary dentition reported so far. Jorgenson suggested physical interruption of a dental lamina as a causative factor of oligodontia as seen in Oro-facial-digital syndrome.^22^ Niemineu et al suggested deletion of MSX 1 gene which is essential for normal oral and tooth development.^23^ Studies have shown a strong correlation between agenesis of primary teeth with agenesis of corresponding permanent teeth^24^ which correlates with the present case. If there is agenesis in the primary dentition and not in the permanent dentition the etiological factor cannot be a defect in the downgrowth of dental lamina. If there is agenesis of both dentitions, it may be due to ectodermal mucosal defect. Stones^17^ reported that oligodontia is associated with dental abnormalities, such as reduced size of teeth and abnormalities of enamel and delayed eruption of teeth.^18^ This case needs to be followed up to determine the correlation of agenesis of both dentitions and variation in shape and structure of developed tooth. Oligodontia requires interdisciplinary management depending upon the age of the patient. The treatment for this abnormality can be challenging and includes removable partial denture, fixed partial denture and overdentures.^15,16^ The choice is dependent on the age of the patient, number of teeth present, condition of the remaining teeth and the treatment cost. The rationale for the use of removable partial denture in this case was acceptable cost, easy adjustment during growth, restoration of vertical dimension, and easy replacement of missing teeth. Between 3 and 6 years, removable partial denture is recommended keeping in view the growing age of the patient. For children between 7 and 12 years, rigid or fixed prosthesis can be used with caution teeth.^16^ In this case, because of the severe atrophy of the alveolar ridge and the age of the patient, dental implants were not suitable at this time. The use of dental implants in future may be a possibility. The patient will be monitored after every 3 months to determine the need to change the removable partial denture. This will be a transitional period during which the patient's response to her prosthesis will be evaluated. Further follow-up for evaluating the role of multiple missing primary teeth in the development of dentofacial structures needs to be evaluated. The treatment guidelines suggested in this paper are an attempt to discuss the options available to the pediatric dentist for oral rehabilitation of oligodontia.

## CONCLUSION

The pediatric dentist can make a significant contribution to the overall development and well being of a child with oligodontia. Early detection and rehabilitation of children with oligodontia will go a long way in helping them interact normally and integrate them with their peers and society.
